# Local Tranexamic Acid Infiltration for Prophylactic Hemorrhage Control in Vaginal Hysterectomy: Double-Blinded Randomized Placebo-Controlled Trial

**DOI:** 10.1007/s00192-025-06497-0

**Published:** 2026-01-14

**Authors:** Yair Daykan, Rina Tamir Yaniv, Yael Yagur, Gal Cohen, Hadar Gluska, Michal Ovadia, Merav Sharvit, Nissim Arbib, Ron Schonman, Zvi Klein

**Affiliations:** 1 Department of Obstetrics and Gynecology, Meir Medical Center, Kfar Saba, Israel; 2 Gray Faculty of Medical & Health Sciences, Tel Aviv University, Tel Aviv, Israel; 3 Mayanei HaYeshua Medical Center, Bnei Brak, Israel

**Keywords:** Blood loss, Local infiltration, Randomized controlled trial, Safety, Tranexamic acid, Vaginal hysterectomy

## Abstract

**Introduction and Hypothesis:**

Tranexamic acid, an antifibrinolytic agent with a well-established safety profile, promotes hemostasis by inhibiting fibrin degradation. In recent years, intravenous tranexamic acid has gained wide acceptance as a prophylactic antihemorrhagic agent in surgical settings. However, data on its local administration remain limited. This study was designed to evaluate whether prophylactic local infiltration of tranexamic acid into cervical and vaginal tissues before surgery could reduce intraoperative blood loss.

**Methods:**

A double-blind, randomized, placebo-controlled trial was conducted at a tertiary medical center between April 2022 and December 2024. Sixty women undergoing benign vaginal hysterectomy were randomized (1:1) to receive either 1 g TXA (in 10 ml saline) or placebo via local cervical/vaginal infiltration 5 min pre-incision. The primary outcome was delta hemoglobin (ΔHb; pre- to postoperative hemoglobin change). Outcomes were compared using appropriate tests (*t* test or Mann–Whitney *U*, as indicated) with α = 0.05. Ethical approval was obtained from our institutional review board.

**Results:**

Sixty patients were enrolled. Baseline characteristics of the two groups were comparable. The mean hemoglobin reduction was 1.48 ± 0.73 g/dl in the tranexamic acid group versus 1.27 ± 1.02 g/dl in controls (*p* = 0.36). Estimated blood loss, transfusion rates, and operative parameters did not differ significantly. Mucosal dissection was significantly easier in the tranexamic acid group (86.7% vs 40.0%, *p* < 0.01). No thromboembolic events occurred.

**Conclusion:**

Local prophylactic tranexamic acid did not reduce total blood loss. Vaginal hysterectomy remains a safe procedure with minimal bleeding.

## Introduction

Vaginal hysterectomy is a widely performed surgical procedure, particularly common owing to the high prevalence of uterine prolapse and other pelvic organ support defects in the aging female population [1, 2]. It remains the preferred approach for women with symptomatic uterine prolapse, offering excellent safety and efficacy profiles. Large studies demonstrate that vaginal hysterectomy is associated with low intraoperative complication rates, short stay, and a low rate of serious adverse events [3–6].

Tranexamic acid (TXA) is an antifibrinolytic agent widely used to treat or prevent excessive bleeding in various clinical settings [7], including heavy menstrual bleeding, trauma, and surgery [8]. It works by inhibiting the activation of plasminogen to plasmin, thereby preventing the breakdown of fibrin clots and promoting hemostasis. TXA is available in oral, intravenous, and topical formulations, and is generally well tolerated, with rare side effects such as visual disturbances or thromboembolic events. Its safety and efficacy have made it a cornerstone in the management of hemorrhagic conditions, including the reduction of postpartum [9, 10] and perioperative blood loss, including cesarean delivery [6].

The prophylactic use of TXA in an intravenous administration in cesarian deliveries [11] and various types of hysterectomy has been shown to reduce overall blood loss and the incidence of significant bleeding [12–14]. Studies have found that TXA is effective in reducing the need for blood transfusions and reoperations owing to postoperative hemorrhage without significantly increasing the risk of adverse events.

Local infiltration or the topical use of TXA has shown promise in reducing perioperative bleeding across various surgeries, especially in the orthopedic field [15–17]. It effectively decreases blood loss, hematoma formation, and the need for drains, even in patients on antithrombotic therapy owing to minimal systemic absorption [18, 19].

Given the demonstrated efficacy and safety of local TXA use in reducing bleeding in other medical fields, our aim was to evaluate whether local infiltration of TXA prior to vaginal hysterectomy effectively decreases intraoperative blood loss.

## Materials and Methods

This double-blinded, randomized, placebo-controlled trial was conducted at a tertiary medical center in Israel between April 2022 and December 2024, adhering to the Consolidated Standards Of Reporting Trials 2010 guidelines for reporting randomized trials [20].

Patients were recruited consecutively 1–2 days prior to an elective vaginal hysterectomy surgery and all surgeries were done during the morning hours. All patients scheduled for vaginal hysterectomy and anterior repair with or without posterior wall repair or midurethral sling insertion who agreed to participate in the study were included and provided signed informed consent. We used the Pelvic Organ Prolapse Quantification classification for pelvic organ prolapse.

Eligible participants were adult patients (over 18 years of age) scheduled for vaginal hysterectomy for benign indications. Exclusion criteria included known hypersensitivity to TXA, coagulopathy, active thromboembolic disease, and known malignancy.

A study protocol was written prior to starting the trial and approved by the Ethics Committee. All surgeries were performed by two subspecialized urogynecology consultants with extensive experience in pelvic floor reconstructive procedures.

After a detailed explanation, eligible patients who agreed to participate in the study signed a consent form. Participants were randomly assigned in a 1:1 ratio to receive either local infiltration of TXA or placebo at the start of surgery. Participants were randomized for the study and the control group using designated software (Randomizer for clinical trials, iOS app version 2.0; Medsharing, Paris, France).

Prophylactic cephalosporin was administered to all patients within 60 min before skin incision, based on the American College of Obstetricians and Gynecologists recommendations.

The intervention group received 1 g of TXA diluted in 10 ml of 0.9% sodium chloride, whereas the control group received 10 ml of 0.9% sodium chloride alone. The study drugs were prepared in identical syringes by pharmacy staff not involved in patient care or outcome assessment, ensuring blinding of both the surgical team and participants.

In the intervention group, 1 g of TXA (in 10 ml saline) was infiltrated into the tissues at the cervicovaginal junction approximately 5 min before the first incision. The surgeon injected the solution in a circumferential manner: about 2–3 ml was injected into the anterior cervical stroma and vaginal submucosa, 2–3 ml into the posterior aspect, and the remainder divided between the lateral (3 and 9 o’clock) positions around the cervix. Injections were made to a depth of roughly 1 cm using a 21-gauge needle to ensure that the solution spread into the submucosal vascular plexus. The placebo group received an identical volume (10 ml of 0.9% saline) with the same technique and timing. This method ensured uniform local distribution of the study solution before surgical dissection began.

Point rescue—in the event of unexpected bleeding during surgery that requires pharmacological intervention, the operating surgeon requested to unblind the treatment allocation. At this point, the patient was withdrawn from the study and was not included in the final analysis. It should be noted that up to 2 g of TXA can be safely administered without concern for side effects; therefore, there is no contraindication to giving an additional dose in the case of increased bleeding.

For the assessment of intraoperative bleeding, a complete blood count, including hemoglobin level, was obtained preoperatively for all patients, and was repeated on the morning after surgery to further assess the extent of blood loss and was calculated as delta hemoglobin (ΔHb). A collection bag was affixed beneath the surgical site to allow for precise measurement of blood loss. At the end of the procedure, the surgeon evaluated the amount of bleeding both subjectively and by weighing the blood collection bag. As per the study protocol, predefined adverse events (including thromboembolic complications) were monitored. All patients were observed for any thromboembolic event during their in-hospital recovery and were followed for 30 days postoperatively (including a routine follow-up visit approximately 4–6 weeks after surgery) to capture any delayed complications.

### Data Collection

The primary outcome was the change in hemoglobin concentration (ΔHb) measured preoperatively and on the 1 st postoperative day. All data regarding patient demographics, intraoperative details, and perioperative laboratory results were prospectively collected. Ethical approval was obtained from the institutional review board prior to study initiation, and all participants provided written informed consent.

### Ethics Approval

The study was approved by the Medical Center Ethics Committee in September 2020, approval number 0354-19-MMC. The study was approved by the local institutional review board.

### Statistical Analysis

The primary outcome was defined as the change in hemoglobin levels (ΔHb) measured before and after surgery within each of the study groups. Categorical variables were analyzed using either the Chi-squared test or Fisher’s exact test, depending on expected cell counts. Continuous variables were first assessed for normality using the Shapiro–Wilk test. This test concluded that body mass index (BMI), hemoglobin, prothrombin time (PT), international normalized ratio (INR), and fibrinogen had normal distribution, whereas maternal age, parity, uterine weight, hematocrit, platelets, and partial thromboplastin time (PTT) did not follow a normal distribution. Variables exhibiting a normal distribution were presented as mean ± standard deviation (SD) and were compared using independent-samples *t* tests, whereas variables not following a normal distribution were presented as median and interquartile range (IQR) and were compared using the Mann–Whitney *U* test. A two-sided *p* value of < 0.05 was considered statistically significant. All statistical analyses were performed using SPSS software, version 29.0 (IBM, Armonk, NY, USA).

### Sample-Size Calculation

We calculated the power analysis based on the known ΔHb reduction during hysterectomies—0.84 g/dl (SD ±0.6) [12] - and the assumption that local administration of TXA can achieve a lower decrease. To determine the required sample size for comparing hemoglobin drop between standard hysterectomy and hysterectomy with TXA, we conducted an a priori sample-size calculation based on a 40% reduction in ΔHb in the TXA group (mean ΔHb ~0.5 g/dl vs 0.84 g/dl in controls) with 80% power and α = 0.05. This 40% relative reduction was chosen based on prior studies suggesting that TXA could reduce blood loss by ~20–30% in hysterectomy; we selected a slightly larger effect size to ensure clinical relevance. This yielded a required sample of 30 patients per arm (60 total).

## Results

**Fig. 1:**
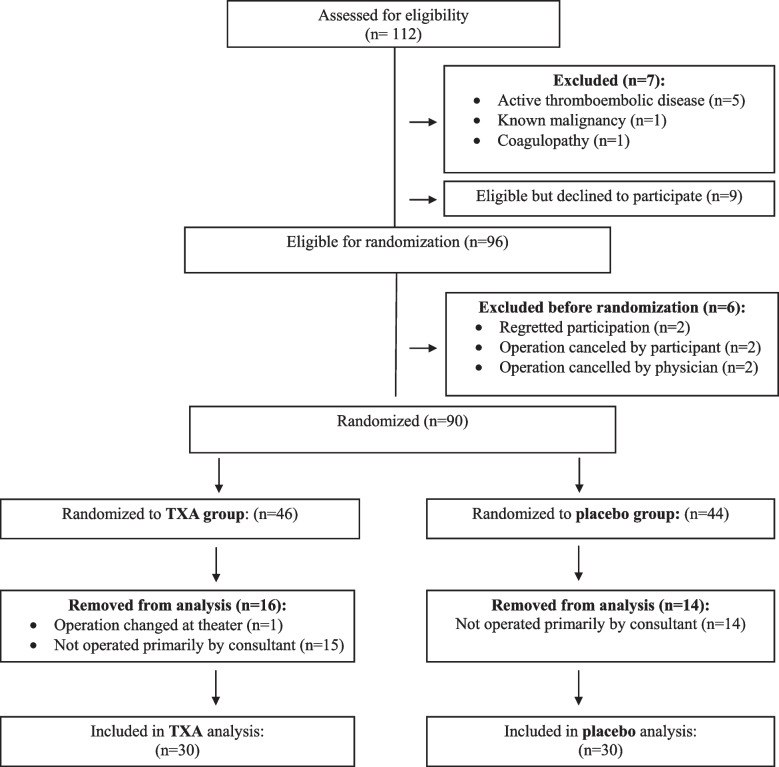
Flowchart describing the study population

During the study period, 112 women scheduled for vaginal hysterectomy were assessed for eligibility. Sixteen were excluded (7 did not meet inclusion criteria, 9 declined participation), leaving 96 eligible. Six were excluded before randomization (2 regretted participation, 2 canceled their operation, and 2 had their operation canceled by the physician). Overall, 90 were randomized (46 TXA, 44 placebo). After exclusions for surgical or operator reasons, 60 women (30 in each group) completed the study and were included in the final analysis. The flowchart of patient recruitment and participation is presented in Fig. 1.

Baseline demographic and clinical characteristics of the two groups were comparable, except for a higher proportion of patients with a history of cesarean delivery in the TXA group (20%). No significant differences were noted in the demographic characteristic such as age, BMI, parity, or concomitant surgical procedures (Table 1).

**Table 1:** Demographic characteristics of the study groups

Variable		Tranexamic acid (*n* = 30)	Sodium chloride (*n* = 30)	*p* value
Maternal age (years)		68.5 (50–73.75)	67 (57.8–75.3)	0.43
Smoking		1 (3.3)	2 (6.7)	>0.99
BMI (kg/m^2^)		27.6 ± 3.9	26.2 ± 4.3	0.24
History of cesarean delivery		6 (20)	2 (6.6)	0.25
Parity		4 (2–5)	4 (3–4)	0.94
History of vaginal repair		1 (3.3)	0 (0.0)	>0.99
Stage of uterine prolapse	Median	3 (2–3)	3 (2–3)	0.21
	0	0 (0.0)	0 (0.0)	>0.99
	1	0 (0.0)	0 (0.0)	>0.99
	2	8 (26.7)	12 (40.0)	0.27
	3	18 (60.0)	16 (53.3)	0.60
	4	4 (13.3)	2 (6.7)	0.67
Stage of cystocele	Median	3 (3–3)	3 (2–3)	0.37
	0	0 (0.0)	0 (0.0)	>0.99
	1	0 (0.0)	0 (0.0)	>0.99
	2	6 (20.0)	8 (26.7)	0.54
	3	20 (66.7)	20 (66.7)	>0.99
	4	4 (13.3)	2 (6.7)	0.67
Stage of rectocele	Median	1 (0–2)	1 (0–2)	0.79
	0	14 (46.7)	12 (40.0)	0.60
	1	8 (26.7)	9 (30.0)	0.77
	2	5 (16.7)	9 (30.0)	0.22
	3	1 (3.3)	0 (0.0)	>0.99
	4	2 (6.7)	0 (0.0)	0.49

Data are presented as number (%); mean ± standard deviation; or median (interquartile range) as appropriate

Prolapse staging: modified POP-Q, International Urogynecological Association/International Continence Society Joint Report on the Terminology for Pelvic Organ Prolapse

*BMI* body mass index (kg/m^2^)

### Primary Outcome

The mean ΔHb, which served as the primary outcome, was slightly greater in the TXA group (1.48 ± 0.73 g/dl) than in the control group (1.27 ± 1.02 g/dl), but this difference was not statistically significant (*p* = 0.36, 95% CI –0.67 to 0.25; see Table 3).

Preoperative hemoglobin levels were similar between the TXA and control groups (12.8 ± 1.3 vs 12.6 ± 1.1 g/dl, *p* = 0.52, 95% CI −0.82 to 0.43). Postoperative hemoglobin levels also showed no difference (11.3 ± 1.3 g/dl in both groups, *p* = 0.96, 95% CI −0.67 to 0.25).

**Fig. 2:**
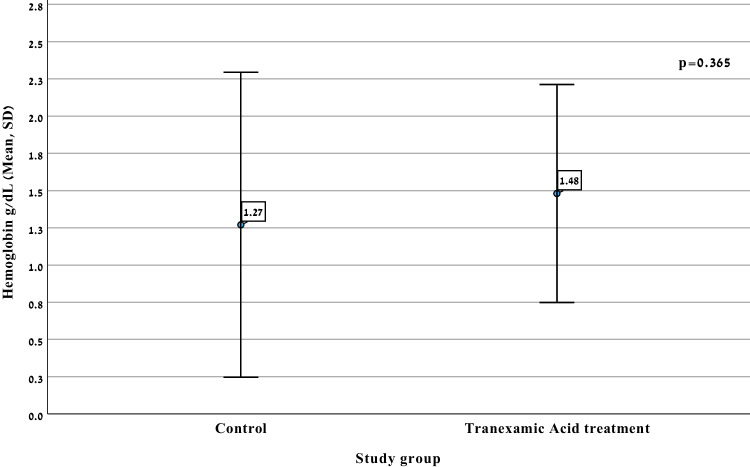
Distribution of hemoglobin change (ΔHb) pre- and postsurgical procedure

**Fig. 3:**
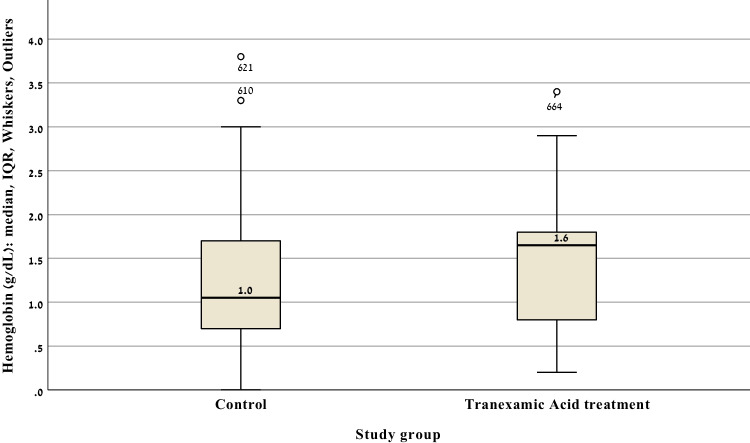
The box represents the interquartile range (IQR), the horizontal line within the box indicates the median, and whiskers extend to the most extreme values within 1.5 × IQR. Circles denote mild outliers. ΔHb values reflect the change in hemoglobin concentration from baseline to the postoperative measurement

Figure 2 illustrates the distribution of hemoglobin change (ΔHb) before and after surgery in both the TXA and the control groups. The graph displays individual data points along with a central tendency marker (likely mean ± standard deviation or median with interquartile range). Although the TXA group appears to exhibit a slightly greater mean reduction in hemoglobin compared with the control group, the spread of values shows substantial overlap. The variability, particularly in the control group, seems higher, reflected by a broader distribution of ΔHb values. A box plot of Δ hemoglobin distribution in the study groups is presented in Fig. 3.

No cases of bleeding >500 ml occurred in the TXA group, compared with one case (3.3%) in the placebo group (*p* > 0.99).

### Preoperative Prolapse Characteristics

Preoperative pelvic organ prolapse stages were similar between the TXA and control groups in all compartments (see Table 1 for detailed stage distributions). No significant differences were observed in prolapse stage or severity across groups.

The stage of uterine prolapse was most commonly stage 3 in both groups (53.3% in the TXA group vs 60.0% in the controls, *p* = 0.60). In terms of anterior compartment (cystocele) prolapse, stage 3 was the most frequent finding in both groups, affecting 66.7% of patients.

Regarding the posterior compartment (rectocele), stage 0 rectocele was documented in 40.0% of the TXA group and 46.7% of the controls (*p* = 0.60).

### Operative and Postoperative Assessments

Operative parameters of the two groups were comparable (Table 2). Median uterine weight did not differ (TXA 55 g vs placebo 66 g, *p* = 0.30). Rates of concomitant procedures (e.g., posterior repair, midurethral sling) were also similar. Notably, surgical dissection was subjectively easier in the TXA group: 86.7% of TXA cases were rated as having “easy” mucosal dissection versus 40.0% in the controls (*p* < 0.01). Despite this, there were no significant differences in estimated blood loss and actual blood collection during surgery between the two groups. No thromboembolic events (e.g., deep vein thrombosis or pulmonary embolism) were observed in either group during hospitalization or the 30-day follow-up period. Postoperative complications, including urinary retention, hematoma, need for blood transfusion, or re-hospitalization, were rare and occurred at similar rates across both groups.

**Table 2:** Operative and postoperative assessment of study groups

Variable		Tranexamic acid (*n* = 30)	Sodium chloride (*n* = 30)	*p* value
Concomitant bilateral salpingo-oophorectomy/salpingectomy		4 (13.3)	1 (3.3)	0.35
Concomitant posterior repair		6 (20)	7 (23.3)	0.75
Concomitant midurethral sling		13 (43.3)	14 (46.7)	0.79
Mucosal dissection difficulty	1	26 (86.7)	12 (40.0)	<0.01
	2	4 (13.3)	17 (56.7)	<0.01
	3	0 (0.0)	1 (3.3)	>0.99
Uterine weight (g)		55 (45–81)	66 (46–95)	0.30
Bleeding estimation (ml)		110 (50–300)	100 (100–213)	0.78
Actual bleeding (ml)		150 (74–300)	150 (85–200)	0.88
Bleeding above 500 ml		0 (0.0)	1 (3.3)	>0.99
Urinary retention		1 (3.3)	0 (0.0)	>0.99
Hematoma/abscess		0 (0.0)	0 (0.0)	>0.99
Need for blood infusion		0 (0.0)	1 (3.3)	>0.99
Re-hospitalization		1 (3.3)	1 (3.3)	>0.99
Re-operation in light of a complication		0 (0.0)	1 (3.3)	>0.99

Data are presented as number (%); mean ± standard deviation; or median (interquartile range) as appropriate

Mucosal separation difficulty—1 = easy separation, 2 = moderate separation, 3 = difficult separation

### Laboratory Findings

Postoperative laboratory values showed no significant group differences. Table 3 provides full blood count and coagulation data.

**Table 3:** Laboratory findings among the study groups

Variable	Tranexamic acid (*n* = 30)	Sodium chloride (*n* = 30)	*p* value	95% confidence interval
Hemoglobin before surgery (g/dl)	12.8 ± 1.3	12.6 ± 1.1	0.53	−0.82 to 0.43
Hemoglobin after surgery (g/dl)	11.3 ± 1.3	11.3 ± 1.3	0.97	−0.64 to 0.67
Delta hemoglobin	1.48 ± 0.73	1.27 ± 1.02	0.36	−0.67 to 0.25
Hematocrit before surgery	40.1 (35.2 to 42.0)	36.3 (38.1 to 41.3)	0.36	NA
Hematocrit after surgery	35.9 (31.2 to 37.9)	33.9 (31.9 to 37.0)	0.37	NA
Delta hematocrit	4.4 (2.9 to 5.5)	3.8 (2.3 to 7.0)	0.76	NA
Platelets before surgery	236,000 (195,000 to 279,000)	250,000 (216,000 to 276,000)	0.50	NA
Platelets after surgery	218,000 (185,000 to 255,000)	216,000 (186,000 to 273,000)	0.97	NA
Delta platelets	12 (−5 to 28)	22 (6 to 47)	0.13	NA
PT (%)	113.2 ± 15.9	106.9 ± 17.4	0.15	−15.00 to 2.41
PT SEC	10.4 ± 0.5	10.6 ± 0.8	0.19	−0.13 to 0.60
PTT	25 (24 to 26)	25 (24 to 27)	0.97	NA
INR	0.96 ± 0.1	0.99 ± 0.1	0.18	−0.01 to 0.06
Fibrinogen	368 ± 95	322 ± 92	0.09	−101.73 to 8.26

Data are presented as number (%); mean ± standard deviation; or median (interquartile range) as appropriate

*PT* prothrombin time, *Seconds* , *INR* international normalized ratio, *PTT* partial thromboplastin time

Coagulation profiles (PT, PTT, INR) and fibrinogen levels were also evaluated. Although mean fibrinogen levels were higher in the TXA group (368 ± 95 mg/dl vs 322 ± 92 mg/dl), the difference did not reach statistical significance (*p* = 0.09, 95% CI −101.73 to 8.26). PT in seconds and percentage activity, as well as INR and PTT, were similar in the two groups.

All other evaluated laboratory parameters did not differ significantly (Table 3).

## Discussion

This double-blinded, randomized, placebo-controlled trial evaluated the efficacy of local TXA infiltration in reducing blood loss during elective vaginal hysterectomy. There was no significant difference in the primary outcome, change in hemoglobin levels (ΔHb), between the two groups (1.48 ± 0.73 vs. 1.27 ± 1.02 g/dl, *p* = 0.36). No significant differences were observed in estimated or measured blood loss, hematocrit change, or coagulation parameters. Mucosal dissection was rated significantly easier in the TXA group (*p* < 0.01), although there was no impact on bleeding. Overall, prophylactic local infiltration of TXA did not reduce perioperative blood loss and did not offer clinical benefit in routine vaginal hysterectomy.

Vaginal hysterectomy is among the most commonly performed gynecological procedures and indicated mainly for pelvic organ prolapse [2]. Numerous studies [12–14, 21] have evaluated the use of intravenous (IV) TXA as a prophylactic measure to reduce intraoperative bleeding during hysterectomy, demonstrating that IV TXA is both effective and safe. Meta-analyses and randomized trials [12, 13, 22] suggest that IV TXA could reduce blood loss by approximately 20–30%, with no associated increase in thromboembolic complications [11, 23].

With IV TXA established as being effective, recent studies have explored whether topical (local) administration of TXA may offer similar hemostatic benefits [16, 17, 19, 24, 25]. This question is particularly relevant for vaginal hysterectomy, where the surgical field allows direct local infiltration. However, many of the published studies [26–28] on TXA in the context of hysterectomy included a heterogeneous mix of surgical approaches; abdominal, laparoscopic, and vaginal, thereby limiting the ability to draw specific conclusions regarding its efficacy in vaginal hysterectomy, where baseline blood loss may differ substantially between techniques. There are currently no randomized controlled trials evaluating local administration of TXA in vaginal hysterectomy specifically for the prophylaxis of bleeding. Our study is among the first to address this gap, focusing solely on vaginal hysterectomy and using locally administered TXA.

Nandi and John [14] reported a mean reduction of approximately 130 ml in intraoperative blood loss with IV TXA compared with placebo in vaginal hysterectomies. However, it is important to recognize the limitations of estimating blood loss intraoperatively, as these values can be highly variable and imprecise owing to factors such as swab saturation and pooled blood in the surgical field. In contrast to prior studies that relied on estimated blood loss [21, 26, 27], we selected ΔHb as our primary outcome owing to its greater reliability and relevance in clinical decision making. Notably, in our cohort, the mean difference in ΔHb between the local TXA and control groups was only 0.21 g/dl, corresponding to an estimated blood loss difference of ~150 ml, which was not statistically significant (*p* = 0.88). This suggests that although TXA may modestly reduce bleeding (ΔHb), such a reduction may not be clinically relevant unless it translates into meaningful outcomes such as reduced transfusion rates.

It is also important to note that even when statistical differences in bleeding or hemoglobin levels are found, these may lack clinical significance if they do not influence key endpoints such as the need for blood transfusion or postoperative recovery. Therefore, transfusion requirement remains a more robust and actionable clinical outcome.

The route of TXA administration has been the subject of several comparative studies. For example, Sallam and Shady [28] randomized women undergoing abdominal hysterectomy to receive IV TXA, topical TXA, or placebo. Both IV and topical TXA significantly reduced blood loss compared with placebo, and no difference was found between the two active treatment groups, suggesting the non-inferiority of topical TXA. Similarly, Mitra et al. [26] compared IV and topical TXA and found no significant difference in total blood loss (312 ± 106 ml vs 325 ± 90 ml respectively; *p* = 0.66), further supporting the notion that local TXA may be as effective as systemic administration.

These findings are consistent with research in other surgical disciplines. A meta-analysis by Montroy et al. [29] reviewed 67 trials across various surgical specialties and found no significant differences in blood loss or transfusion rates between IV and topical TXA. Similarly, a medical review [18] in plastic surgeries concluded that local TXA reduces bleeding to a similar extent to IV use, with the added advantage of minimal systemic absorption and potentially fewer systemic side effects.

One potential rationale for local TXA is to minimize systemic exposure, which in theory could reduce thrombotic risk. However, IV TXA has consistently demonstrated a strong safety profile, even in large gynecological studies. In a multicenter trial [12] involving 332 patients undergoing hysterectomy, no thromboembolic events were reported in either the TXA or the placebo group. Likewise, in our study, there were no differences in postoperative deep vein thrombosis or pulmonary embolism, reaffirming the safety of TXA.

We believe that the lack of advantage observed in our trial with local TXA infiltration compared with placebo may be explained by two main factors. First, the overall blood loss in vaginal hysterectomy is typically modest; therefore, even if a reduction occurs, it may not reach clinical significance. Second, locally infiltrated TXA may not sufficiently enter the systemic circulation to influence total bleeding.

Interestingly, surgeons reported that tissue dissection was subjectively easier in the TXA group. One hypothesis is that the local infiltration of TXA solution itself may have created a mild hydro-dissection effect, separating tissue planes and thereby facilitating the surgical dissection. The TXA could also have reduced small-vessel oozing, improving visualization in the operative field. The clinical significance of this subjective ease is therefore unclear, although it could conceivably contribute to better surgeon comfort in difficult cases.

### Clinical Implications

This study provides valuable evidence suggesting that local infiltration of TXA during vaginal hysterectomy does not confer a significant reduction in perioperative blood loss compared with placebo. As the first RCT focused on local TXA in vaginal hysterectomy, our findings suggest that routine prophylactic use of locally administered TXA might be unlikely to provide additional benefit in the context of elective benign vaginal hysterectomy—a procedure already associated with minimal blood loss and low transfusion rates. Nonetheless, these data are hypothesis generating for certain higher-risk groups. Patients with a larger uterus, extensive concomitant repairs, prior pelvic surgery, or those on anticoagulant therapy may have higher baseline bleeding risk; TXA (especially intravenous) may confer benefits in such cases. Our negative results should be interpreted with caution in these contexts, and further studies targeting these subpopulations are warranted. Interestingly, the subjective ease of dissection in the TXA group may imply some benefit in surgical handling or visualization. For the urogynecological surgeon, a smoother dissection (potentially due to tissue turgor from the injection) could mean a slightly faster or less arduous surgery, even if blood loss is unchanged.

### Strengths and Limitations

A major strength of this study is its rigorous double-blinded, randomized, placebo-controlled design, which minimizes bias and enhances the validity of the findings. The study was adequately powered based on a priori calculations. All surgeries were performed by experienced urogynecology consultants, ensuring procedural consistency. Additionally, both subjective and objective measures of blood loss were used, including ΔHb and measured blood collection, to provide a comprehensive assessment.

However, this study is not without limitations. The modest sample size (*n* = 60) may have limited our ability to detect very small but clinically relevant differences in blood loss—in other words, a lack of statistical significance could partly reflect insufficient power rather than a true absence of effect. It must be emphasized that the naturally low blood loss of vaginal hysterectomy inherently limits the potential to observe a difference due to any hemostatic intervention. In a setting where bleeding is minimal, even an effective drug may not demonstrate a large absolute benefit.

Furthermore, being conducted at a single tertiary center may affect the generalizability of the results to broader surgical populations or other health care settings. The relatively long recruitment time reflects our intention to maintain procedural consistency, as all operations were performed by only two urogynecological surgeons to limit inter-surgeon variability and minimize bias. Last, the lack of long-term follow-up precludes conclusions regarding delayed complications or the potential long-term benefits of TXA use.

## Conclusions

In this double-blinded, randomized, placebo-controlled trial, local infiltration of TXA during elective vaginal hysterectomy did not significantly reduce perioperative blood loss compared with placebo. Vaginal hysterectomy alone is generally associated with low blood loss; therefore, the routine use of local TXA may not be warranted based on our findings. However, given the single-center design and relatively small sample, these findings should be interpreted with caution. This study is hypothesis generating and underscores the need for further research. In particular, larger multicenter trials comparing local versus systemic (IV) TXA in vaginal hysterectomy—especially in higher-risk scenarios—are warranted to definitively determine if TXA can benefit those subsets of patients.

## Data Availability

Data will be made available from the corresponding author upon reasonable request.

## Abbreviations

ΔHb: Delta hemoglobin change
IV: Intravenous
TXA: Tranexamic acid
